# Lithium and long-term cognitive outcomes in bipolar disorder and early dementia: a systematic review

**DOI:** 10.1017/S1092852926100996

**Published:** 2026-06-09

**Authors:** Rosalyn Deng, Alisa Shargorodsky, Kayla Teopiz, Christine E. Dri, Sabrina Wong, Gia Han Le, Yang Jing Zheng, Roger S. McIntyre

**Affiliations:** 1 Queen’s University Faculty of Health Sciences, Canada; 2 Brain and Cognition Discovery Foundation, Canada; 3 Department of Human Biology, University of Toronto, Canada; 4 University of Toronto Institute of Medical Science, Canada; 5 Poul Hansen Family Centre for Depression, University Health Network, Canada; 6 Department of Pharmacology, University of Toronto, Canada; 7 Department of Psychiatry, University of Toronto, Canada

**Keywords:** Lithium, cognition, bipolar disorder, mild cognitive impairment, Alzheimer’s disease, randomized controlled trial

## Abstract

Cognitive impairment is a major determinant of disability in bipolar disorder (BD) and a defining feature of both mild cognitive impairment (MCI) and Alzheimer’s disease (AD). Lithium, a first-line maintenance treatment for BD, is implicated in neuroprotective mechanisms including glycogen synthase kinase-3β inhibition, amyloid and tau modulation, and neurogenesis promotion. The overarching aim of this systematic review is to evaluate the long-term effects of lithium on cognition across BD, MCI, and early-to-moderate AD using randomized controlled trial (RCT) evidence. Online databases were searched from inception through May 2025 for RCTs reporting lithium’s effect on cognitive outcomes in BD, MCI, or early-to-moderate AD with ≥8 weeks of follow-up. Risk of bias was assessed using the Cochrane RoB 2 tool. Eight RCTs met the inclusion criteria, ranging from 10 weeks to 3 years in duration. Across four BD trials, lithium did not exhibit consistent improvement or worsening on composite cognitive scores. Three of four MCI/AD trials reported attenuated global cognitive deterioration with low-dose lithium, especially when exposure was ≥12 months. Methodological limitations included small sample sizes, exploratory endpoints, and variable measures for cognitive function as well as lithium strategies. Lithium demonstrates preliminary signals of slower cognitive decline in MCI/AD. Available evidence suggests lithium has neutral effects on cognitive impairment in BD. Future adequately powered RCTs with cognition as a primary endpoint, functional measures, and biomarker outcomes are warranted to clarify lithium’s role as a maintenance treatment in psychiatric disorders and its potential neuroprotective effects in neurodegenerative diseases.

## Introduction

Alzheimer’s disease (AD) and related dementias affect over 57 million individuals worldwide and are among the leading causes of disability and need for long-term care.^1^ ^,^^2^ Mild cognitive impairment (MCI) is a heterogeneous clinical syndrome representing a transitional state between normal aging and dementia.^3^ ^,^ ^4^ Many patients with MCI remain stable, however, it is estimated that 10%–15% of affected individuals progress to dementia annually.^5^

Bipolar disorder (BD) affects approximately 1%–2% of adults globally and is highly associated with cognitive deficits and reduced psychosocial and occupational outcomes.^6^ ^–^^11^ Convergent evidence indicates that persons with BD are at an increased risk of developing MCI or early dementia in later life.^12^ ^–^^17^ Although BD, MCI, and dementia differ in etiology and clinical trajectory, they share overlapping features of cognitive dysfunction, particularly in memory, attention, and executive function.^18^ ^–^^21^ Notably, replicated evidence suggests that the foregoing conditions may also share common pathways related to neuroinflammation, mitochondrial dysfunction, and impaired neuroplasticity.^22^ ^–^^26^

Preliminary research investigating the effect of lithium, a first-line pharmacologic treatment for individuals with BD, supports the hypothesis that cognitive impairment in BD, MCI, and early dementia may share overlapping pathophysiologic pathways.^27^ ^–^^31^ For example, a case–control study of adults with BD reported that treatment with lithium may reduce the prevalence of AD at follow-up.^12^ Observational studies in BD further suggest that sustained lithium exposure may be associated with a lower risk of dementia compared with other mood stabilizers or no treatment, although these findings may be confounded by illness severity and treatment adherence.^6^ ^,^^9^ ^,^^21^ Mechanistically, experimental and clinical studies have identified a potential neuroprotective role of lithium, possibly preventing the onset of AD.^32^ Proposed mechanisms include the inhibition of glycogen synthase kinase-3β (GSK-3β), modulation of amyloid and tau pathology, promotion of neurogenesis, and reduction of oxidative stress.^33^ ^–^^35^ While tau and amyloid deposition are central to AD and MCI, BD-related neuroprogression is linked to the aforementioned neurotrophic pathways, suggesting that lithium’s cognitive effects across disorders likely operate through distinct mechanisms.

In contrast, randomized controlled trials (RCTs) evaluating the effect of lithium in persons with BD have largely reported no significant change in cognitive performance, although it is noted that cognition has not typically been assessed as a primary outcome measure.^36^ ^–^^39^ In older adults with amnestic MCI (aMCI) or early-to-moderate AD, several small randomized and longitudinal trials have reported slower cognitive decline and favorable changes in disease-related biomarkers in persons treated with low-dose lithium. However, the foregoing results remain inconsistent due to variability in dose, treatment duration, serum levels, and cognitive assessments.^28^ ^,^^40^ ^–^^46^ Collectively, these findings provide the rationale for examining whether lithium’s potential neuroprotective mechanisms, via GSK-3β inhibition in MCI/AD and modulation of inflammatory and neurotrophic pathways in BD, lead to cognitive benefits across distinct clinical contexts. This apparent discrepancy between observational and RCT data also underscores the importance of interpreting lithium’s cognitive impact within each disorder, while cautiously exploring potential areas of overlap.

Extant literature has primarily examined lithium and cognition in persons with BD and persons with MCI or AD separately.^47^ ^,^^48^ A recent systematic review by Sabtiari et al. (2025) summarized lithium’s cognitive effects across mixed study designs; however, the heterogeneity of included methodologies and the scarcity of controlled long-term data limited their ability to draw conclusions.^49^ Hereto, no analysis to our knowledge has delineated evaluation exclusively to RCTs, while assessing long-term cognitive outcomes across both psychiatric and neurocognitive populations collectively. A comparative synthesis may therefore clarify lithium’s cognitive profile and inform prevention and treatment strategies, given shared mechanistic targets and the central role of cognition in prognosis and functional independence.^50^

This systematic review aimed to synthesize available RCT data to assess the association between lithium and improvement or stabilization of long-term cognitive outcomes in persons with BD, MCI, or early-to-moderate AD. Specifically, this review aimed to assess the long-term effect of lithium on cognitive outcomes beyond acute treatment effects (defined a priori as ≥8 weeks of follow-up). We also aimed to explore potential moderators such as dose, serum concentration, duration of exposure, and mood state. This review adopted a comparative framework that examines BD and MCI/AD in parallel, rather than assuming a single shared mechanism across disorders, to highlight both shared and disorder-specific patterns of cognitive change with lithium.

## Methods

### Search strategy

This review was conducted in accordance with the Preferred Reporting Items for Systematic Reviews and Meta-Analyses (PRISMA) 2020 guidelines.^51^ A comprehensive literature search was performed using online databases PubMed, Scopus, ClinicalTrials.gov, and Ovid MEDLINE, from inception through May 13, 2025. Search terms included BD, MCI, AD, lithium, and various outcome-related terms. The Boolean search string was: (“bipolar disorder” OR “Alzheimer*” OR “mild cognitive impairment” OR “dementia”) AND (“lithium”) AND (“relapse” OR “recurrence” OR “conversion” OR “decline” OR “cognitive” OR “function*” OR “longitudinal” OR “progress*”). The full search string is described in Supplementary Table S1. Reference lists of included studies were also screened manually. Only English-language, human studies were considered.

### Study selection and eligibility criteria

Study selection was managed using Covidence software.^52^ After the automatic removal of duplicates and non-RCTs by Covidence, two reviewers (R.D., A.S.) independently screened the remaining records. Screening was conducted first by title and abstract, followed by full-text review to confirm eligibility. Disagreements were resolved by discussion until a consensus was reached. Articles deemed relevant by both reviewers were included for data extraction and analysis.

Studies were included if they were RCTs investigating the effect of lithium (any formulation or dose, as monotherapy or adjunctive) in adults (≥18 years) with BD, MCI, or early-to-moderate AD, and reported at least one validated cognitive outcome. Long-term outcomes were defined a priori as ≥8 weeks to exclude acute pharmacologic studies and capture trials assessing cognitive change beyond immediate treatment effects. This threshold was selected pragmatically, as there is currently no consensus regarding the duration of exposure required to evaluate potential neuroprotective or disease-modifying effects. Only retrievable, full-text articles in English were considered. Eligible comparators included placebo or active treatments. Studies were excluded if they were nonrandomized, case series or reports, reviews, meta-analyses, animal studies, or trials in populations with severe dementia or mixed diagnoses without separable data. See Table 1 for full eligibility criteria.

**Table 1.** Eligibility Criteria Table 1. long description.

Inclusion criteria	Exclusion criteria
RCTs, including secondary analyses reporting cognitive outcomes Adults (≥18 years) with BD I/II, MCI, or early-to-moderate AD Lithium as intervention (any formulation or dose; monotherapy or adjunctive) Comparator: placebo or active control At least one validated cognitive outcome at follow-up (e.g., MMSE, ADAS-Cog, CAMCOG, TMT, MCCB, MOS-Cog) Long-term follow-up is defined a priori as ≥8 weeks post-baseline Full text available in English; human studies	Nonrandomized designs (observational, quasi-experimental), case series/reports, narrative reviews, systematic reviews, meta-analyses Populations with severe dementia or mixed diagnoses without separable data No lithium arm or lithium not isolatable from co-interventions No comparator group No cognitive outcome reported, or only subjective cognition Follow-up <8 weeks Full-text unavailable or not in English; animal or pediatric studies

Table outlining the criteria used to determine which studies were included in the review. This table describes the eligibility criteria used to select studies for inclusion in the review. It outlines the requirements related to study participants, interventions, study design, outcomes, and other factors considered when determining whether a study met the inclusion criteria.

### Data extraction

Data items were prespecified before screening. Extracted information included (1) author(s) and year; (2) country; (3) study population and sample size; (4) trial design; (5) lithium serum levels and regimen; (6) comparator; (7) follow-up duration; (8) cognitive outcomes and instruments; and (9) main cognition findings. Data extraction was performed by one reviewer (R.D.). Study characteristics are summarized in Table 2.

**Table 2.** Study Characteristics of Included RCTs Table 2. long description.

Author(s) and Year	Country	Study population and sample size	Trial design	Lithium regimen and serum levels	Comparator	Follow-up duration	Cognitive outcomes and instruments	Main cognition findings
Weisler RH et al., 2011	International, 128 centers in 15 countries in Asia, Europe, Central and South America, and the United States	Population: Adults with BD I recently stabilized after an acute manic episode; predominantly euthymic at cognitive testing Sample size: 1172 Arms: 404 quetiapine; 364 lithium; 404 placebo	Multicenter, randomized, double-blind, placebo-controlled, parallel-group trial	Regimen: Monotherapy Serum level: 0.6–1.2 mEq/L (mean 0.63 mEq/L [SD 0.45] ITT; 0.77 mEq/L [SD 0.13] per-protocol) Formulation: Capsules identical to quetiapine/placebo	Active comparator (quetiapine [300–800 mg/d, mean (SD) 546 (173) mg]), placebo	Baseline, weeks 4, 8, 12, 20, 28, 40, 52, 68, 84, and 104, or whenever early termination occurred	MOS-Cog, TMT	Global Cognition (MOS-Cog):—Lithium produced a small improvement compared with placebo - Quetiapine also improved scores relative to placebo Processing Speed/Attention (TMT-A):—Lithium showed no significant change compared with placebo—Quetiapine showed no significant change compared with placebo Executive Function (TMT-B):—Lithium showed no significant difference from placebo - Quetiapine showed a nonsignificant trend toward better performance
Johnstone EC et al., 1990	United Kingdom	Population: Adults with BD I receiving adjunctive lithium during maintenance treatment Sample size: 40 Arms (BD subgroup): 5 lithium + amitriptyline; 8 lithium + placebo	Randomized, double-blind, placebo-controlled trial	Regimen: Adjunctive or monotherapy Serum level: 0.5–0.9 mEq/L	Active comparator (adjunct [amitriptyline (mean 87.7 (36.6))] versus monotherapy [placebo])	Monthly assessment, up to 3 years	Digit symbol substitution (WAIS), CFQ	- No significant differences in cognitive test scores between groups—No significant changes in cognitive scores over time in all groups—No relationship between mood/depression ratings and cognitive test results—Bipolar sample too small for robust conclusions, but no signal for cognitive benefit or harm from lithium
Daglas R et al., 2016	Australia	Population: Adults with BD I following a first manic episode; transitioned to euthymia during follow-up Sample size: 61 Arms: 31 quetiapine; 30 lithium	Randomized, controlled, open-label, parallel-group trial. Note: Secondary analysis of neuropsychological outcomes	Regimen: Monotherapy Serum level: 0.8–1.0 mmol/L (acute phase); 0.6–0.8 mmol/L (maintenance phase)	Active comparator (quetiapine)	Baseline, months 3, and 12	TMT, Digit Symbol Coding, Cogstate (Detection, Identification, One-card Learning, One-card Maze, GMLT), Span Forward, Go/no-go, Attention Network Task (ANT), Rey Auditory Verbal Learning Test (RAVLT), Digit Span Backward, Verbal Fluency (FAS, Animal Naming), Stroop Test	Processing Speed:—No significant differences between lithium and quetiapine across TMT-A, Digit Symbol Coding, Cogstate Detection reaction time Attention:—No significant group differences on Span Forward, Go/No-Go, total errors, or Cogstate Identification tasks Sustained Attention:—No significant differences between groups across all ANT indices, including alerting, executive control, and congruent/incongruent performance Verbal Learning and Memory:—No significant group differences on Rey Auditory Verbal Learning Test (Trials 1, short delay, long delay) at 3 or 12 months—No significant differences on Cogstate One-Card Learning or One-Card Maze tasks Working Memory:—No significant group difference on Digit Span Backward Verbal Fluency:—Lithium group showed a significantly greater improvement in verbal fluency (FAS total correct) at 3 (*p* = 0.025) and 12 months (*p* = 0.017) compared with quetiapine Executive Functioning/Flexibility:—No significant group differences on Trail Making Test B, Stroop, Groton Maze
Zhuo C et al., 2022	China	Population: Adults with BD II during depressive or hypomanic episodes Sample size: 209 total (149 drug-naïve BD II; 60 healthy controls) Arms (for cognition analyses): 25 lithium (depression phase); 26 lithium (mania/hypomania phase)	Randomized, controlled, open-label, parallel-group trial	Regimen: Monotherapy Dose: 600 mg/day (Serum levels not reported)	For depression (DP): lithium versus lithium+LTG, LTG alone, VPA + LTG For hypomania (MA): lithium versus VPA	Baseline, 12 weeks	MATRICS Consensus Cognitive Battery (MCCB) domains: Processing speed, Attention/vigilance, Working memory, Verbal learning, Visual learning, Reasoning/problem solving, Social cognition, Composite cognition score	Processing Speed:—Lithium monotherapy (DP/Li, MA/Li) did not significantly alter scores from baseline—Significant declines were observed in DP/LTG, DP/Li + LTG, DP/LTG + VPA, and MA/VPA Attention/Vigilance:—No significant change in DP/Li or MA/Li—Significant post-treatment deterioration in DP/LTG, DP/Li + LTG, DP/LTG + VPA, and MA/VPA Working Memory:—Lithium groups (DP/Li, MA/Li) remained stable without decline—Significant decreases occurred in DP/LTG, DP/Li + LTG, DP/LTG + VPA, and MA/VPA Verbal Learning:—Lithium groups showed no significant change—Declines were significant in LTG and VPA, with the greatest worsening in DP/LTG + VPA and MA/VPA Visual Learning:—DP/Li patients showed a small, non-significant trend toward improvement (*p* = 0.050)—LTG and VPA declined significantly across both DP- and MA-onset cohorts Reasoning/Problem Solving:—No deterioration in lithium monotherapy groups—Significant declines in DP/LTG, DP/Li + LTG, DP/LTG + VPA, and MA/VPA Social Cognition:—Lithium was neutral or slightly improved (DP/Li, *p* = 0.048)—LTG and VPA groups demonstrated worsening, particularly DP/LTG + VPA and MA/VPA Composite Cognition:—No significant changes in DP/Li or MA/Li, indicating cognitive stability—Marked deterioration in DP/LTG, DP/Li + LTG, DP/LTG + VPA, and MA/VPA
Forlenza OV et al., 2011	Brazil	Population: Adults with aMCI Sample size: 45 Arms: 23 lithium; 22 placebo	Randomized, double-blind, placebo-controlled trial	Regimen: Monotherapy Serum level: 0.25–0.5 mmol/L Formulation: Lithium carbonate tablets	Placebo	Baseline, 12 months	ADAS-Cog, CAMCOG, TMT	Global Cognition (ADAS-Cog):—Lithium remained stable, indicating prevention of cognitive decline—Placebo showed significant worsening, demonstrating a between-group advantage for lithium Working Memory (CAMCOG):—Lithium remained stable across follow-up—Placebo showed a significant decline (*p* = 0.04) Processing Speed (TMT-A):—Both lithium (*p* = 0.08) and placebo (*p* = 0.13) showed small, nonsignificant improvements—No significant between-group differences Executive Function (TMT-B):—No significant changes in either lithium or placebo—No between-group differences
Forlenza OV et al., 2019	Brazil	Population: Adults with aMCI Sample size: 61 Arms: 31 lithium; 30 placebo	Randomised, double-blind, placebo-controlled trial (2 years), followed by single-blind 2-year extension (total 4 years); stratified randomisation by age/education	Regimen: Monotherapy Serum level: 0.25–0.5 mEq/L Mean serum level: 0.39 mEq/L (SD 0.08) during double-blind phase; 0.41 mEq/L (SD 0.10) during extension phase Formulation: Lithium carbonate tablets	Placebo	Baseline, months 12, and 24	ADAS-Cog, Sequence of Letters and Numbers, Delayed Recall, Figure Recall, TMT	Global Cognition (ADAS-Cog):—Lithium had a smaller worsening over 2 years (+0.83)—Placebo had a significantly greater worsening (+1.80, *p* < 0.05) Working Memory (Sequence of Letters and Numbers)—Lithium and placebo both had a nonsignificant decline, and the difference between groups was not statistically significant Memory: Delayed Recall - Lithium had improvement over time (*p* < 0.001) - Placebo experienced a decline Memory: Figure Recall - Lithium showed improvement (*p* = 0.02) - Placebo had a nonsignificant change Processing Speed/Attention (TMT-A)—Lithium showed a small improvement—Placebo demonstrated a significant worsening (*p* < 0.05) Executive Function (TMT-B)—Lithium showed no significant change—Placebo showed no significant change
Nunes A et al., 2013	Brazil	Population: Adults with moderate AD Sample size: 113 Arms: 58 lithium; 55 placebo	Randomized, double-blind, placebo-controlled trial	Regimen: Monotherapy Dose: 300 μg/day (microdose) Formulation: Lithium gluconate or carbonate capsule	Placebo	Baseline, every 3 months for 15 months	MMSE	Global Cognition (MMSE) - Lithium group showed higher MMSE scores beginning at trimester 1 (*p* < 0.01)—Between-group difference widened progressively through trimester 5 (*p* < 0.001)
Hampel H et al., 2009	Germany	Population: Adults with mild to moderate AD Sample size: 71 Arms: 31 lithium; 38 placebo	Multicenter, randomized, single-blind (assessors blinded), placebo-controlled trial	Regimen: Monotherapy Serum level: 0.5–0.8 mmol/L	Placebo	Baseline, 10 weeks	ADAS-Cog, MMSE	Global Cognition (MMSE):—Lithium group showed a small, nonsignificant decrease over follow-up—Placebo group showed a similarly small, nonsignificant change with no between-group difference. Global Cognition (ADAS-Cog):—Lithium group remained essentially stable across follow-up - Placebo group showed a slight numerical worsening but no significant group difference

Abbreviations: AD, alzheimer’s disease; ADAS-Cog, alzheimer’s disease assessment scale – cognitive subscale; ANT, attention network test; BD, bipolar disorder; CAMCOG, cambridge cognitive examination; CFQ, cognitive failures questionnaire; cogstate, computerized neurocognitive test battery (detection, identification, one-card learning, one-card maze, groton maze learning test); FAS, verbal fluency test – letter fluency (F, A, S); GMLT, groton maze learning test; ITT, intention-to-treat; LTG, lamotrigine; M(SD), Mean (Standard Deviation); MA, mania/hypomania; MCCB, MATRICS consensus cognitive battery; MMSE, mini-mental state examination; MOS-Cog, medical outcomes study – cognitive functioning scale; NS, nonsignificant; RAVLT, rey auditory verbal learning test; SD, standard deviation; SE, standard error; TMT, trail making test; VPA, valproate; WAIS, wechsler adult intelligence scale (digit symbol substitution).

*Note:* Summary of the main characteristics of the randomized controlled trials included in the review. This table provides an overview of the randomized controlled trials included in the review. It summarizes important study details such as the authors, sample size, participant characteristics, interventions, duration of the study, and outcomes assessed. Serum lithium units are reported as in the original publications (mmol/L or mEq/L).

Given the nature of the extracted data, a quantitative meta-analysis was not performed due to substantial clinical and methodological heterogeneity across trials, including differences in study populations, lithium dosing, follow-up duration, and cognitive outcome measures.

### Quality assessment

**Figure 1.**
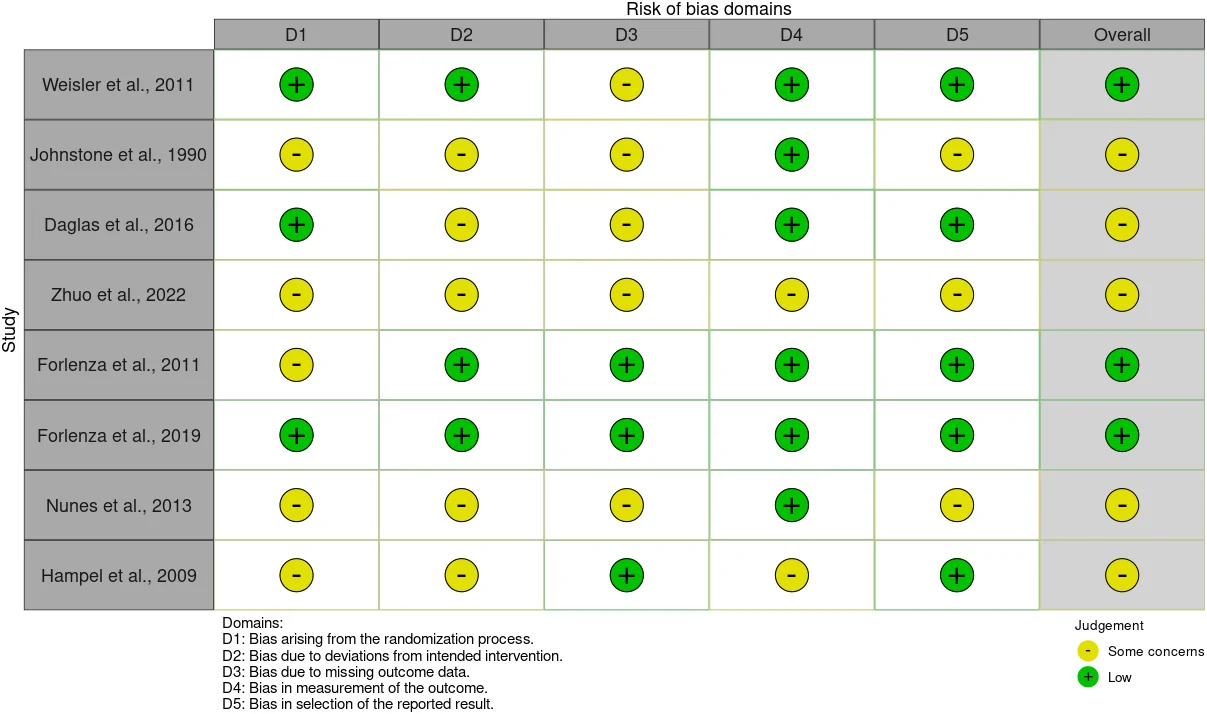
Risk of bias assessment of included RCTs. Figure summarizing the risk of bias across the included randomized controlled trials. Figure 1. long description.

Two reviewers (R.D., A.S.) independently assessed the quality of all included trials using the Cochrane Risk of Bias tool for randomized trials (RoB 2).^53^ Each study was evaluated across domains of randomization, adherence to intended interventions, missing data, outcome measurement, and selective reporting. Domain-level judgments are presented in Figure 1.

## Results

### Search results and selection

**Figure 2.**
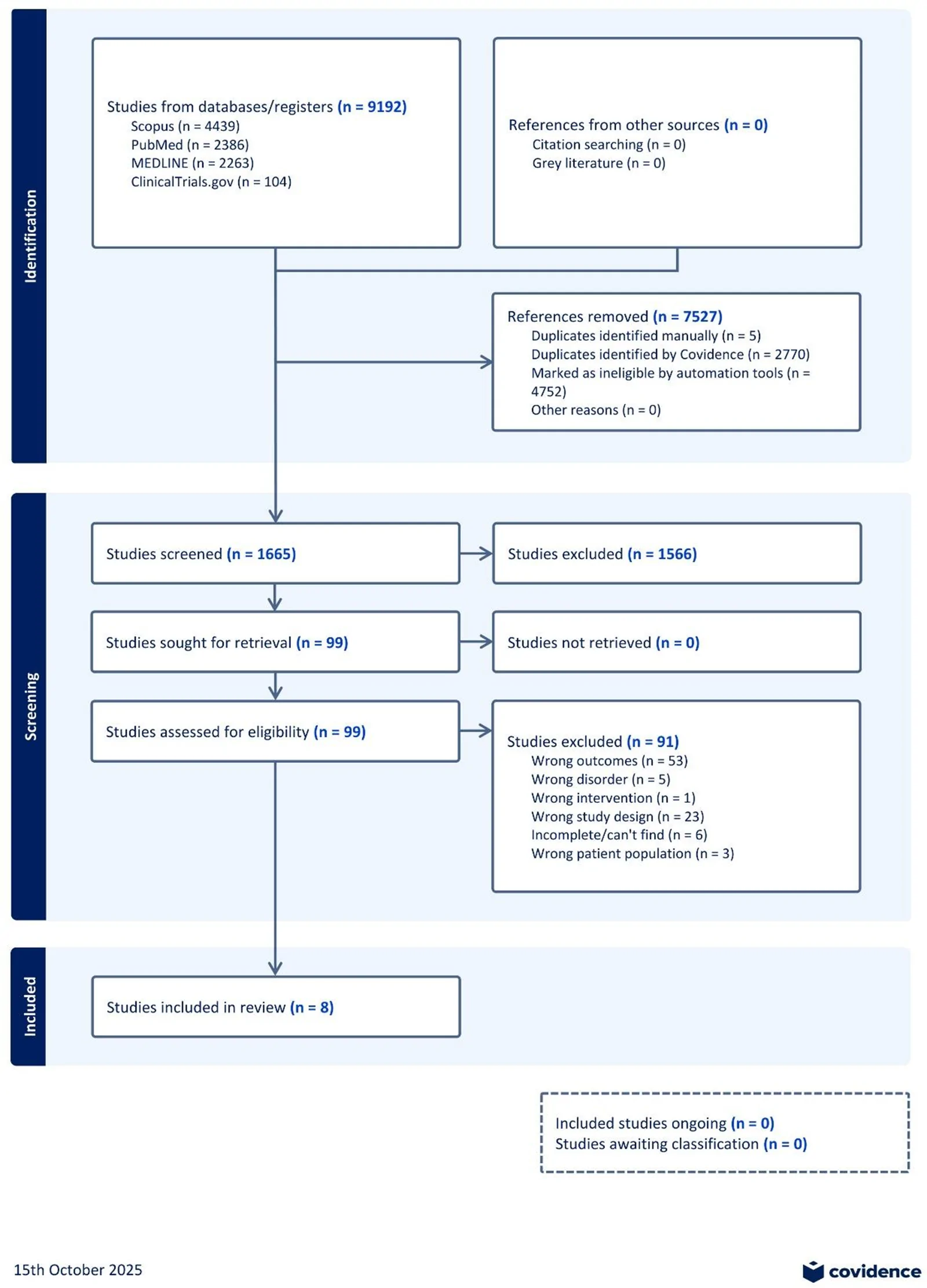
PRISMA flow diagram of literature search. Flow diagram showing how studies were identified and selected for the review. Figure 2. long description.

The initial database search generated 9192 records. After removal of duplicates (*n* = 2770) and application of Covidence’s automated non-RCT exclusions (*n* = 4752), 1670 records remained for title and abstract screening. Of these, 99 studies underwent full-text review, yielding eight eligible RCTs for data extraction and qualitative synthesis. The most common reasons for exclusion at the full-text stage were incomplete study or unavailable full text (*n* = 6), outcomes not aligned with the review focus (*n* = 53), wrong study design (*n* = 23), and wrong disorder or patient population (*n* = 9). The screening process is further detailed in the PRISMA flow diagram (Figure 2).

### Characteristics of included studies

The eight included trials examined the effects of lithium on long-term cognitive outcomes in two clinical groupings: BD (*n* = 4) and neurocognitive disorders (aMCI and early-to-moderate AD) (*n* = 4).^36^ ^–^^39^ ^,^^41^ ^,^^42^ ^,^^45^ ^,^^54^ Study designs ranged from small, single-site pilot trials to large multicenter RCTs. Sample sizes ranged from 40 to 1172 participants, with follow-up periods between 10 weeks and 3 years. BD trials primarily enrolled younger or middle-aged adults (mean ages 23–39 years), with female representation ranging from 36% to 57%.^36^ ^–^^39^ In contrast, MCI/AD trials recruited older adults (mean ages 70–77 years), with 49%–64% female participants across studies.^41^ ^,^^42^ ^,^^45^ ^,^^54^

In the included studies, dosage and frequency of lithium varied by sample population, with standard therapeutic serum concentrations (0.5–1.2 mEq/L) typically used in BD trials, while low-dose levels (as low as 300 μg/day) were tested in MCI/AD trials.^36^ ^–^^39^ ^,^^41^ ^,^^42^ ^,^^45^ ^,^^54^ Comparators included placebo in all 4 MCI/AD trials and a range of active-control groups in BD studies (e.g., quetiapine [target 300–800 mg/d], lamotrigine [dose not reported], valproate [dose not reported], and amitriptyline [mean 87.7 mg/d]).^36^ ^–^^39^ ^,^ ^41^ ^,^^42^ ^,^^45^ ^,^^54^ Cognitive outcomes were assessed using validated tools, including the Mini-Mental State Examination (MMSE), Alzheimer’s Disease Assessment Scale-Cognitive Subscale (ADAS-Cog), Cambridge Cognitive Examination (CAMCOG), Trail Making Test (TMT), Medical Outcomes Study Cognitive Functioning Scale (MOS-Cog), and the MATRICS Consensus Cognitive Battery (MCCB).^36^ ^–^^39^ ^,^^41^ ^,^^42^ ^,^^45^ ^,^^54^

### Methodological quality assessment

Risk of bias varied across studies, as shown in Figure 2. The large multicenter RCT by Weisler et al. (2011) was generally methodologically robust but had notable attrition (around 47%); however, dropout rates were well-described and evenly distributed across arms.^36^ It is noted, however, that cognition remained an exploratory outcome in the foregoing study.^36^

Forlenza et al. (2011, 2019) conducted two double-blind placebo-controlled trials evaluating lithium’s effect on cognitive function in persons with aMCI with over a year of follow-up and predefined cognitive endpoints.^41^ ^,^^42^ The main limitation of the 2011 trial was incomplete reporting of the allocation concealment procedures, which was later addressed in the 2019 study.^41^ ^,^^42^

Daglas et al. (2016) was a single-blind trial with blinded cognitive assessors, introducing potential for performance or measurement bias.^38^ Similarly, Hampel et al. (2009) was a single-blind trial with a 10-week treatment duration and cognition as an exploratory outcome.^45^

Greater methodological limitations were noted in smaller and older trials. Johnstone et al. (1990) lacked clear documentation of randomization, used adjunctive therapy, and had high dropout (62.5%) and incomplete follow-up.^37^ Zhuo et al. (2022) was open-label and lacked clear blinding and randomization, although missing data were minimal.^39^ Nunes et al. (2013) was limited by a small sample size and incomplete reporting of randomization and missing data handling.^54^

### Cognitive outcomes

#### Bipolar disorder

Across four RCTs (*n* = 1422), lithium demonstrated a consistently neutral effect on cognition relative to placebo and active comparators.^36^ ^–^^39^ Global measures of cognition, such as MOS-Cog and MCCB, remained stable over 10 weeks to 2 years, with no evidence of progressive decline.^36^ ^–^^39^ Similarly, no between-group differences were observed in executive function and attention (as measured by the TMT and MCCB subtests) between persons receiving lithium or placebo.^36^ ^–^^39^

However, modest improvement in verbal fluency was observed in lithium-treated participants, recruited at their first episode of mania, at both three (*p* = 0.025) and 12 months (*p* = 0.017) compared to quetiapine.^38^ Across most other cognitive domains (i.e., processing speed, attention and vigilance, working memory, verbal learning, visual learning, reasoning and problem solving, social cognition, and composite cognition), no significant group differences emerged.^38^ ^,^^39^ In contrast, valproate comparators were associated with cognitive worsening, whereas lithium-treated participants did not experience significant cognitive decline.^39^ Overall, available evidence from RCTs indicates that maintenance treatment with lithium in persons with BD did not impair cognition but may also offer a selective benefit in early illness.

#### Mild cognitive impairment and Alzheimer’s disease

Across four RCTs evaluating lithium treatment in persons with MCI or AD (*n* = 290), lithium attenuated global cognitive decline when administered at low-dose levels (300 μg-0.5 mmol/L) for ≥12 months.^41^ ^,^^42^ ^,^^45^ ^,^^54^ On MMSE and ADAS-Cog, lithium groups showed either stabilization or slower rates of deterioration compared with placebo.^41^ ^,^^42^ ^,^^54^ These effects were most commonly reported in trials lasting 12–24 months in duration and were sometimes accompanied by functional stabilization.^41^ ^,^^42^ ^,^^54^ For instance, maintenance or slower progression of difficulties in daily living activities (e.g., managing finances, dressing, bathing, and other basic self-care), as measured by the Clinical Dementia Rating-Sum of Boxes (CDR-SoB) score, was reported in lithium-treated patients compared to placebo groups.^41^ ^,^^42^

In shorter interventions (i.e., <12 weeks), neither the lithium nor placebo groups experienced significant changes in cognitive outcomes; however, the lithium group’s scores tended to remain stable or decline less than placebo, though the difference was not statistically significant.^45^ Thus, cognitive protection appears most apparent with low-range dosing and extended follow-up.

## Discussion

We identified eight RCTs that met eligibility criteria evaluating lithium’s long-term effects on cognition in BD, MCI, or early-to-moderate AD, revealing population-specific and design-dependent patterns. The studies included in this review provide preliminary evidence that lithium may be associated with slower cognitive decline in MCI/AD. However, the evidence of lithium’s effect on cognition in persons with BD remains sparse and heterogeneous, with most trials in BD being underpowered or not designed to assess cognition as a primary outcome. As a result, available evidence from the included trials requires careful consideration of dose, age, disease stage, and duration of exposure when interpreting the possible effects of lithium on cognitive function in these patient populations. Given substantial differences in etiology, age of onset, and outcome sensitivity across disorders, these findings should be interpreted as parallel, side-by-side results rather than evidence of a unified mechanistic process.

Among older adults with MCI/AD, three of the four RCTs included in our review demonstrated that lithium slowed cognitive decline relative to placebo, as assessed by global cognition measures including the MMSE and ADAS-Cog, with the strongest effects in trials lasting at least 12 months and at low doses.^41^ ^,^^42^ ^,^^45^ ^,^^54^ However, it is noted that the included trials were small and heterogeneous in dose and duration, limiting interpretation of the findings. Furthermore, it should be considered that the two trials conducted by Forlenza et al. may have included overlapping participants in the sample.^41^ ^,^^42^

Separately, the four included trials evaluating the effect of lithium in BD showed that cognitive performance remained stable over varied time periods ranging from 10 weeks to 3 years, with one trial demonstrating modest improvements in verbal fluency; though this result must be interpreted cautiously given multiple comparisons.^36^ ^–^^39^ Overall, the available evidence suggests that lithium treatment did not increase or decrease cognitive decline in persons with BD. These results are relevant to longstanding clinical concerns over lithium-related cognitive toxicity. It is noted that BD trials typically employ therapeutic-range serum concentrations of lithium, which may be associated with cognitive side effects; however, the current data suggest that no cognitive decline was observed across the included studies.

In persons with BD, therapeutic-range lithium was consistently identified as cognitively neutral with limited domain-specific benefits, whereas in older adults with MCI/AD, low-dose lithium was associated with slower decline on various cognitive scales. Cognitive endpoints differed by patient population: BD studies emphasized executive and attention measures, whereas MCI/AD trials prioritized global decline scales. Cognitive battery sensitivity also differed across studies, with MCI/AD trials relying primarily on global screening measures sensitive to neurodegenerative decline, whereas BD trials employed multi-domain neuropsychological batteries in younger populations with relatively stable cognitive trajectories. The foregoing heterogeneity in outcome measures complicates direct comparison of findings across studies. Despite these constraints, available evidence suggests that lithium is unlikely to harm cognition in BD and may be associated with the stabilization of cognitive outcomes in MCI/AD.

The present findings accord with prior narrative and meta-analytic work. Wingo et al. (2009) conducted a meta-analysis of studies across mixed designs, identifying no evidence of lithium-related cognitive toxicity in BD, specifically, no deficits in memory or attention, though small reductions in immediate verbal learning and psychomotor speed were noted.^27^ However, the foregoing observations may be influenced by cross-sectional and observational data and are confounded by illness duration and polypharmacy.^27^ Additional observational studies have reported a significant reduction of dementia amongst persons living with BD with chronic exposure.^6^ ^,^^21^ In populations with MCI/AD, Matsunaga et al. (2015) reviewed three RCTs, showing that low-dose lithium slowed cognitive deterioration.^33^ They reported that lithium was associated with a modest but statistically significant slowing of cognitive decline overall, with subgroup analyses indicating a nonsignificant effect in MCI alone but a trend toward benefit in AD.^33^ Our review thus builds upon prior work by synthesizing additional evidence from recently published RCTs and provides a comparative framework across disorders. A recent review by Sabtiari et al. (2025) emphasized considerable methodological heterogeneity.^49^ By isolating RCT evidence, our synthesis refines the evidence base and clarifies remaining gaps and limitations.

Cognitive deficits in memory, executive function, and attention are central to both BD and MCI/AD, and increasing evidence points to overlapping neurobiological mechanisms, such as impaired neuroplasticity, GSK-3β dysregulation, oxidative stress, and impaired neurotrophic signaling.^55^ ^–^^58^ Lithium’s proposed biological mechanisms that may confer neuroprotective effects, including GSK-3β inhibition, enhanced neurotrophic signaling, mitochondrial stabilization, and reduced oxidative stress, uniquely position it to target these pathways.^35^ ^,^^59^ ^–^^64^ In MCI/AD populations, these mechanisms may manifest as stabilization of cerebrospinal fluid (CSF) biomarkers such as phosphorylated tau and Aβ_42_, as reported in the Forlenza et al. trials.^41^ ^,^^42^

However, BD does not share Alzheimer-type tau or amyloid pathology, and BD-related neuroprogression appears linked to cellular resilience and synaptic plasticity.^65^ This mechanistic divergence may partly explain why lithium did not demonstrate either consistent increase or decrease in total cognitive function across BD trials. Importantly, many neurotrophic pathways modulated by lithium require prolonged exposure to produce measurable cognitive or structural effects, raising the possibility that the duration of BD trials was insufficient to detect change.^33^ ^,^^59^ ^,^^61^ ^,^^62^

From a clinical perspective, BD findings suggest that maintenance lithium does not impose a cognitive burden and may offer domain-specific advantages (e.g., verbal fluency). In MCI/AD, longer-duration and low-dose lithium was associated with stabilization on MMSE and ADAS-Cog. While not currently standard of care, consideration of lithium in more research settings, with appropriate renal and thyroid monitoring and biomarker anchoring, is merited.^66^

From a research perspective, emerging evidence suggests that lithium’s potential neuroprotective effects may vary across salt formulations. Recent preclinical and postmortem data indicate that specific formulations, such as lithium orotate, may exhibit differential bioavailability and interactions with amyloid, with implications for neuroprotection.^32^ Accordingly, the findings of the present review should be interpreted in the context of the lithium formulations evaluated in the included trials, and further work is needed to determine whether alternative salts confer distinct cognitive or disease-modifying effects.

There are several methodological limitations that constrain the interpretation of our findings and limit direct cross-disorder comparisons, inherent to the current availability of RCTs. Cognitive outcomes were often secondary, several trials were single-blind or open-label, and sample sizes were limited, especially in older studies, increasing the risk of performance or detection bias, despite the use of blinded raters in the majority of trials. Heterogeneity in population characteristics (e.g., age and diagnostic criteria), lithium dosing strategies (therapeutic vs. microdose and serum levels), cognitive batteries, and follow-up durations precluded meta-analysis, limited precision of effect estimates, and reduced comparability across studies, thereby limiting the strength and generalizability of conclusions. Interpretation of cognitive outcomes should also consider study quality, as several included trials were rated as having “some concerns” across risk-of-bias domains, which may have influenced observed effect estimates, particularly in MCI/AD trials, suggesting potential stabilization of cognitive outcomes. In addition, a number of the BD studies included participants in active or recently remitted mood episodes, complicating attribution of cognitive change to lithium. Generalizability was further restricted by incomplete reporting of sex and ethnoracial composition, as well as geographic clustering in certain regions. Naturalistic cognitive trajectories also differ, with BD typically showing stable or slowly progressive executive deficits versus measurable decline in MCI/AD.^3^ ^,^^7^ ^,^^9^ ^,^^47^ There are a number of methodological considerations that impact the interpretation of the findings in the included BD trials. For example, Zhuo et al. (2022) enrolled younger adults with BD II during active depressive or hypomanic symptoms, which may introduce heterogeneity in producing inherently variable cognitive performance and limit comparability with the euthymic BD I populations.^39^ Furthermore, Weisler et al. (2011) reported an abrupt discontinuation of quetiapine in the lithium treatment arm, which may have transiently affected early assessments of cognition and impacted observations.^36^ The definition of long-term exposure (≥8 weeks) was pragmatically defined and may have influenced study inclusion, as longer-duration trials may be more relevant for evaluating potential disease-modifying effects. In addition, the review protocol was not prospectively registered, which may increase the risk of methodological bias.

There remains a lack of RCTs that investigate the effect of lithium on the neurobiological mechanisms that may underlie the overlapping cognitive deficits in persons with BD and MCI/AD. The majority of included trials lacked mechanistic endpoints, and few included neuroimaging or CSF analyses alongside cognitive assessments. Further research should include adequately powered, double-blinded RCTs, extending beyond 3 years, with cognition and daily function as co-primary outcomes. Harmonization of cognitive batteries spanning executive and memory domains would improve cross-trial comparisons, and dose-finding studies with blinded serum levels are needed to address differences between therapeutic and low-dose regimens. Trials in MCI/AD should incorporate biomarker endpoints, particularly CSF p-tau and amyloid, with evidence of GSK-3β engagement to further investigate disease modification. In parallel, studies in BD should consider older adults, euthymic samples, and longer durations to evaluate potential neuroprotective effects across the lifespan. Methodological guidance from mood disorder cognition task forces can standardize endpoints and enable greater comparative analysis.^43^ ^,^^44^ Investigating lithium’s dose–response across the lifespan and in prodromal dementia phases will help clarify the window of optimized prevention potential.^3^ ^,^^15^

## Conclusion

Across RCTs, lithium was neither consistently pro- nor anti-cognitive in individuals with BD but demonstrated preliminary signals of slower cognitive decline in MCI/AD, particularly in reducing progressive cognitive impairment when used at low-dose levels for extended periods. These findings provide reassurance against longstanding concerns regarding lithium-related cognitive toxicity in BD and highlight its potential as a neuroprotective agent in neurodegenerative disorders. Importantly, these findings arise from distinct clinical contexts and should not be interpreted as a single transdiagnostic effect. Instead, they highlight where the current evidence is too limited to draw definitive cross-disorder conclusions and where future RCTs are more urgently needed. As such, the central implication of this review is the need for adequately powered RCTs with cognition as a primary endpoint and inclusion of functional and biomarker outcomes. The current lack of such trials warrants an opportunity to clarify lithium’s role as a maintenance treatment in psychiatric disorders and to further investigate its potential effects on cognitive decline in neurodegenerative diseases.

## Supporting information

10.1017/S1092852926100996.sm001 Deng et al. supplementary material Deng et al. supplementary material

## Supplementary material

The supplementary material for this article can be found at http://doi.org/10.1017/S1092852926100996.

## Long descriptions

### Table 1. Long description

The table is divided into two main columns: Inclusion criteria and Exclusion criteria.

Inclusion criteria include:

1. R C T s, including secondary analyses reporting cognitive outcomes.

2. Adults greater than or equal to 18 years with B D I or I I, M C I, or early-to-moderate A D.

3. Lithium as intervention in any formulation or dose, monotherapy or adjunctive.

4. Comparator: placebo or active control.

5. At least one validated cognitive outcome at follow-up, such as M M S E, A D A S-Cog, C A M C O G, T M T, M C C B, or M O S-Cog.

6. Long-term follow-up defined a priori as greater than or equal to 8 weeks post-baseline.

7. Full text available in English; human studies.

Exclusion criteria include:

1. Nonrandomized designs such as observational or quasi-experimental, case series or reports, narrative reviews, systematic reviews, and meta-analyses.

2. Populations with severe dementia or mixed diagnoses without separable data.

3. No lithium arm or lithium not isolatable from co-interventions.

4. No comparator group.

5. No cognitive outcome reported, or only subjective cognition.

6. Follow-up less than 8 weeks.

7. Full-text unavailable or not in English; animal or pediatric studies.

Navigate back to Table 1..

### Table 2. Long description

The table consists of nine columns: Author and Year, Country, Study population and sample size, Trial design, Lithium regimen and serum levels, Comparator, Follow-up duration, Cognitive outcomes and instruments, and Main cognition findings.

1. Weisler R H et al., 2011: International study of 1172 adults with B D I. Lithium serum levels 0.6 to 1.2 mEq per L. Findings: Small improvement in global cognition (M O S-Cog) compared to placebo; no significant change in processing speed or executive function.

2. Johnstone E C et al., 1990: U K study of 40 adults with B D I. Lithium serum levels 0.5 to 0.9 mEq per L. Findings: No significant differences in cognitive scores between groups or over time.

3. Daglas R et al., 2016: Australian study of 61 adults with B D I. Lithium serum levels 0.6 to 1.0 mmol per L. Findings: Lithium group showed significantly greater improvement in verbal fluency (F A S) at 3 and 12 months compared to quetiapine; no other significant differences.

4. Zhuo C et al., 2022: Chinese study of 209 adults with B D I I. Lithium dose 600 mg per day. Findings: Lithium monotherapy showed cognitive stability (Composite Cognition), while significant declines were observed in groups taking lamotrigine or valproate.

5. Forlenza O V et al., 2011: Brazilian study of 45 adults with a M C I. Lithium serum levels 0.25 to 0.5 mmol per L. Findings: Lithium remained stable in global cognition (A D A S-Cog), while placebo showed significant worsening.

6. Forlenza O V et al., 2019: Brazilian study of 61 adults with a M C I. Lithium serum levels 0.25 to 0.5 mEq per L. Findings: Lithium showed significantly less worsening in global cognition and improvement in delayed and figure recall compared to placebo.

7. Nunes A et al., 2013: Brazilian study of 113 adults with moderate A D. Microdose lithium 300 micrograms per day. Findings: Lithium group showed significantly higher M M S E scores starting at trimester 1, with the difference widening through trimester 5.

8. Hampel H et al., 2009: German study of 71 adults with mild to moderate A D. Lithium serum levels 0.5 to 0.8 mmol per L. Findings: No significant differences between lithium and placebo in M M S E or A D A S-Cog scores.

Navigate back to Table 2..

### Figure 1. Long description

A matrix table titled Risk of bias domains. The vertical y-axis lists eight studies. The horizontal x-axis lists five domains labeled D 1 through D 5 and an Overall column.

Legend:

* Green circle with a plus sign: Low risk.

* Yellow circle with a minus sign: Some concerns.

Domains:

* D 1: Bias arising from the randomization process.

* D 2: Bias due to deviations from intended intervention.

* D 3: Bias due to missing outcome data.

* D 4: Bias in measurement of the outcome.

* D 5: Bias in selection of the reported result.

Data by Study (D 1, D 2, D 3, D 4, D 5, Overall):

* Weisler et al., 2011: Low, Low, Some concerns, Low, Low, Overall Low.

* Johnstone et al., 1990: Some concerns, Some concerns, Some concerns, Low, Some concerns, Overall Some concerns.

* Daglas et al., 2016: Low, Some concerns, Some concerns, Low, Low, Overall Some concerns.

* Zhuo et al., 2022: Some concerns, Some concerns, Some concerns, Some concerns, Some concerns, Overall Some concerns.

* Forlenza et al., 2011: Some concerns, Low, Low, Low, Low, Overall Low.

* Forlenza et al., 2019: Low, Low, Low, Low, Low, Overall Low.

* Nunes et al., 2013: Some concerns, Some concerns, Some concerns, Low, Some concerns, Overall Some concerns.

* Hampel et al., 2009: Some concerns, Some concerns, Low, Some concerns, Low, Overall Some concerns.

Navigate back to Figure 1..

### Figure 2. Long description

The flowchart is divided into three vertical sections.

1. Identification phase at the top. Two boxes feed into the process. The left box shows Studies from databases/registers n equals 9192, including Scopus n equals 4439, PubMed n equals 2386, M E D L I N E n equals 2263, and ClinicalTrials dot gov n equals 104. The right box shows References from other sources n equals 0. An arrow points to a box on the right indicating References removed n equals 7527, which includes 5 manual duplicates, 2770 Covidence duplicates, and 4752 marked as ineligible by automation tools.

2. Screening phase in the middle. The central flow continues down to Studies screened n equals 1665. An arrow to the right points to Studies excluded n equals 1566. The remaining Studies sought for retrieval n equals 99 leads to Studies not retrieved n equals 0. This leads to Studies assessed for eligibility n equals 99. An arrow to the right points to Studies excluded n equals 91, with reasons including wrong outcomes n equals 53, wrong disorder n equals 5, wrong intervention n equals 1, wrong study design n equals 23, incomplete n equals 6, and wrong patient population n equals 3.

3. Included phase at the bottom. The final central box shows Studies included in review n equals 8. A separate dashed box at the bottom right notes Included studies ongoing n equals 0 and Studies awaiting classification n equals 0.

Navigate back to Figure 2..
