# Supplementary material for: Lithium and long-term cognitive outcomes in bipolar disorder and early dementia: a systematic review
Source: CNS Spectr. 2026 Jun 9;31(1):e18. doi: 10.1017/S1092852926100996 (PMC13732605; doi:10.1017/S1092852926100996)
Supplement: Supplementary file 1 [file S1092852926100996sup001.docx]

**Supplementary Table S1. Full database-specific search strategies**

| Database | Search strategy |
| --- | --- |
| PubMed | ("bipolar disorder"[Title/Abstract] OR "Alzheimer*"[Title/Abstract] OR "mild cognitive impairment"[Title/Abstract] OR "dementia"[Title/Abstract]) AND ("lithium"[Title/Abstract]) AND ("relapse"[Title/Abstract] OR "recurrence"[Title/Abstract] OR "conversion"[Title/Abstract] OR "decline"[Title/Abstract] OR "cognitive"[Title/Abstract] OR "function*"[Title/Abstract] OR "longitudinal"[Title/Abstract] OR "progress*"[Title/Abstract]) Filters applied: Humans; English language |
| Scopus | TITLE-ABS-KEY("bipolar disorder" OR "Alzheimer*" OR "mild cognitive impairment" OR "dementia") AND TITLE-ABS-KEY("lithium") AND TITLE-ABS-KEY("relapse" OR "recurrence" OR "conversion" OR "decline" OR "cognitive" OR "function*" OR "longitudinal" OR "progress*") Filters applied: Language: English; Document types excluded: Review, Letter, Conference Paper, Note, Editorial, Short Survey, Book Chapter, Retracted |
| ClinicalTrials.gov | (bipolar disorder OR Alzheimer OR mild cognitive impairment OR dementia) AND lithium AND (cognitive OR decline OR progression OR function) Filters applied: No filters applied |
| Ovid MEDLINE | (bipolar disorder OR Alzheimer* OR mild cognitive impairment OR dementia).mp. AND lithium.mp. AND (relapse OR recurrence OR conversion OR decline OR cognitive OR function* OR longitudinal OR progress*).mp. Filters applied: Humans; English language |
